# Survey of Expert Opinion on Intelligence: Causes of International Differences in Cognitive Ability Tests

**DOI:** 10.3389/fpsyg.2016.00399

**Published:** 2016-03-23

**Authors:** Heiner Rindermann, David Becker, Thomas R. Coyle

**Affiliations:** ^1^Department of Psychology, Chemnitz University of TechnologyChemnitz, Germany; ^2^Department of Psychology, University of Texas at San AntonioSan Antonio, TX, USA

**Keywords:** intelligence, survey, experts, cross-national differences

## Abstract

Following Snyderman and Rothman (1987, 1988), we surveyed expert opinions on the current state of intelligence research. This report examines expert opinions on causes of international differences in student assessment and psychometric IQ test results. Experts were surveyed about the importance of culture, genes, education (quantity and quality), wealth, health, geography, climate, politics, modernization, sampling error, test knowledge, discrimination, test bias, and migration. The importance of these factors was evaluated for diverse countries, regions, and groups including Finland, East Asia, sub-Saharan Africa, Southern Europe, the Arabian-Muslim world, Latin America, Israel, Jews in the West, Roma (gypsies), and Muslim immigrants. Education was rated by *N* = 71 experts as the most important cause of international ability differences. Genes were rated as the second most relevant factor but also had the highest variability in ratings. Culture, health, wealth, modernization, and politics were the next most important factors, whereas other factors such as geography, climate, test bias, and sampling error were less important. The paper concludes with a discussion of limitations of the survey (e.g., response rates and validity of expert opinions).

## Introduction

Cognitive ability is understood as the ability to think (intelligence), the disposition of knowledge (the store of true and relevant knowledge), and the intelligent use of this knowledge. The current study focuses on international differences in cognitive ability, based on test scores from different countries. International differences in cognitive ability have been estimated using student assessment studies (e.g., TIMSS, PISA, PIRLS; OECD, 2013) and psychometric IQs (Lynn and Vanhanen, 2012)^1^. These international differences in cognitive ability are strongly related to country-level differences in wealth, health, democracy, lifespan, education, innovation, and gross-national product (e.g., Hanushek and Woessmann, 2015; Rindermann et al., 2015).

Differences between countries with the lowest and highest ability levels are large. For example, in TIMSS 2011, 4th grade Yemeni pupils achieved 209 student assessment study (SAS) points, whereas South Korean pupils achieved 587 SAS points. If SAS points are converted to IQ points, the Yemeni would have an IQ of 56 and the Koreans would have an IQ of 113, a difference of 11 years of schooling^2^. Psychometric IQ studies show similar results. For example, Malawi has an estimated IQ of 60, whereas Singapore and Hong Kong have estimated IQs around 108, a difference that translates into *SAS*≈233 and 555 or 16 years of schooling^3^.

Cross-country differences in cognitive ability form geographically contiguous areas with smooth transitions. The lowest ability levels are found in sub-Saharan Africa, the highest levels in East Asia, and moderate to high levels in Europe and other developed countries. The ability levels span a range of 3 *SD*s in IQ. Such a large range has raised questions about the validity, measurement, and causes of the differences (e.g., Hopmann et al., 2007; Wicherts et al., 2010). Discussed causes have included education, modernization, politics, wealth, culture, and evolutionary-genetic factors (e.g., Lynn and Vanhanen, 2012).

Different factors have been stressed for different regions, nations, and cultures. These factors include the quality of educational and support systems (e.g., school health care, families) in Finland (Döbert et al., 2004), low educational levels in Arabian countries (UNDP (United Nations Development Programme) Arab Fund for Economic Social Development, 2003), a culture of effort and achievement in East-Asia (Helmke and Hesse, 2002), health issues in sub-Saharan Africa (Glewwe and Kremer, 2006), and evolutionary-genetic factors in sub-Saharan Africa and East-Asia (e.g., Lynn, 1990; Kanazawa, 2004; Rushton, 2004). Genetic factors have provoked particularly heated disputes, but even the mere description of international ability differences has been contentious (e.g., Hunt, 2012; Coyle et al., 2013; Sternberg, 2013).

One solution to reduce conflict would be to survey experts—scientists who have conducted research on cognitive ability and who have an informed opinion about group differences. Such a solution was adopted in the current study, which examined expert opinions on international ability differences. It is possible that expert opinions may closely approximate the truth. However, majority itself is no sure indicator for truth, especially in a field with zeitgeist or political pressures that favor a particular viewpoint or opinion (e.g., Jussim et al., 2015).

But an expert survey shows two advantages: First, according to the Spearman-Brown prediction formula (Spearman, 1910), increases in the items being analyzed (e.g., test items or expert ratings) will increase the reliability of the final averaged result. The truth value of averaged expert opinions was first described by Galton (1907), who asked stockbreeders and butchers at a cattle market to estimate the weight of an ox. Galton analyzed the estimates and found that the mean value of all results was correct within 1% of the real value. That is, the average opinion of experts was very close to the real answer. The accuracy of expert opinions may extend to the current study, which surveyed experts on international ability differences.

Second, in the current study, data collection procedures were designed to ensure anonymity. The anonymity was implemented to reduce pressure for socially desirable responses, and to increase the likelihood of obtaining honest opinions. Opinions made in anonymity (without fear of retribution) may differ from public appraisals such as those reported in Gottfredson's (1994) “Mainstream Science on Intelligence,” which was signed by more than 50 researchers.

Thirty years ago, Snyderman and Rothman (1987, 1988) surveyed experts on the concept, measurement, and heritability of intelligence, and also on race, class, and cultural differences in intelligence. In the current study, we surveyed experts on the causes of international ability differences. Such differences were not addressed by Snyderman and Rothman, but have received much recent attention (e.g., Wicherts et al., 2010; Hunt, 2012; Lynn and Vanhanen, 2012).

## Methods

### Survey: Expert questionnaire on cognitive ability

To examine expert opinions, we created a new questionnaire (“Expert Questionnaire on Cognitive Ability”). The questionnaire was partly based on the one developed by Snyderman and Rothman (1987, 1988), but also comprised current research topics such as the FLynn effect and international ability differences. Additional questions addressed the definitions of terms and concepts (e.g., intelligence and cognitive ability); validity of tests (e.g., WAIS, SAT, PISA); estimation of genetic and environmental factors; intelligence and the media; genetic testing (e.g., DNA screening); and development of intelligence in global regions and populations. There were 62 main questions with follow-up questions and space for comments. The current study focused on questions concerning the causes of international ability differences.

### Questions

The first general question was, “On causes of cross-national differences, what do you think are the reasons for large differences in cognitive ability and intelligence studies (including PISA, TIMSS etc.)?” This and subsequent questions were followed by the statement, “To address this question, please rate the factors below in importance (it is difficult to clearly distinguish them because they are somewhat connected, so please give only a rough estimate).” There were 15 factors, each rated on a scale from 0 to 100 in 5% steps, with “no answer” also possible. 0% would suggest that the factor explains 0% of international differences and is unimportant; 100% would suggest that the factor explains all of the differences and is highly important. The 15 factors were culture (e.g., religion, traditions), genes, educational quantity, educational quality, wealth, health, geography, climate, politics, modernization, sampling error, test knowledge, discrimination, test bias, and migration.

To obtain additional information, the same questions were asked for the following scientifically or politically important countries and groups:

- Finland: “One of the countries with the highest scores on international assessments (especially PISA) is Finland. Why do you think this is the case?”
- East Asia: “The region with the highest scores on international assessments is East-Asia (Japan, Korea, the Chinas). Why do you think this is the case?”
- sub-Saharan Africa: “The region with the lowest scores on international assessments is sub-Saharan Africa (Nigeria, Kenya or South Africa). Why do you think this is the case?”
- Southern Europe (compared to North-Western-Middle Europe): “Results in Southern Europe (Portugal, Italy or Greece) are lower than results in North-Western-Middle Europe (United Kingdom, France, Netherlands, Finland or Germany). Why?”
- Arabian-Muslim world (compared to North-Western-Middle Europe): “Results in the Arabian-Muslim world (Egypt, Saudi Arabia or Iran) are lower than results in North-Western-Middle Europe (United Kingdom, France, Netherlands, Finland or Germany). Why?”
- Latin America (compared to North America): “Results in Latin America (Mexico, Brazil or Argentina) are lower than results in USA or Canada. Why?”
- Israel (compared to North-Western-Middle Europe): “Results in Israel are lower than results in North-Western-Middle Europe. Why?”
- Jews in the Western world: “Results of Jews in the Western world are at the top. Why?”
- Roma: “Results of Roma (gypsies) in Europe are comparatively low. Why?”
- Muslim immigrants: “Results of immigrants from the Middle East (Arabian and Muslim countries) in the Western world are not at the top. Why?”

Each country or group was rated on the same 15 factors used to evaluate general cross-national differences, using the same scales (0–100), questions, and instructions. In addition, comments could be given for each factor being rated.

### Experts

Notice of the study was emailed to experts who published articles on or after 2010 in journals on intelligence, cognitive abilities, and student achievement. The journals included *Intelligence, Cognitive Psychology, Contemporary Educational Psychology, New Ideas in Psychology*, and *Learning and Individual Differences*. Notice of the study was also emailed to members of the International Society for Intelligence Research (ISIR), and posted to the web site for the International Society for the Study of Individual Differences (ISSID). ISIR and ISSID support intelligence research and host professional conferences with intelligence researchers. Finally, the study was announced at the 2013 ISIR conference in Melbourne, Australia. A total of 1345 people received an email invitation. An expert was defined as a person who had published on cognitive ability or who had attended intelligence conferences and presented research. Compared to Snyderman and Rothman (1988, pp. 46–49), our selection criteria were based more on publications in specific scientific journals and less on membership in scientific organizations. In addition, we used email and a web-based survey rather than traditional mail and paper-pencil surveys.

We received a total of 265 responses from May 2013 to March 2014, at which time the survey was closed. The response rate was 20% of all invitations. The present article focused on cross-national differences and concerned questions toward the end of the survey. These questions were answered by 71 respondents. By comparison, there were 1020 invitations and 661 participants (65%) in the Snyderman and Rothman study.

Respondents were 83% male and 17% female, and had a mean age of 49 years (*SD* = 14.87, *median* = 47 years). Respondents were mainly from the United States (38%), Germany (17%), Scandinavia (9%), the UK and Ireland (8%), Spain (7%), Canada (6%), and Australia/New Zealand (6%). The rest were from Latin America, Austria, Switzerland, Italy, Russia, The Netherlands, Greece, Portugal, and China. Around 20% had at least some self-rated Jewish heritage. The religious background of the respondents' families was about one third Catholic, one third Protestant, and one third with no affiliation. About 6% of respondents were Jewish (religion), and 62% professed no religious affiliation.

Participants worked in the fields of psychology (80%), education (8%), biology (5%), economics (3%), sociology (2%), and physical anthropology (2%). 87% had a Ph.D. All were scientists (i.e., no journalists). Sixty seven percent were tenured faculty, 24% non-tenured faculty, and 6% students. Other participants (about 3%) were not categorized or worked in non-academic research institutes.

The majority of respondents did research on the “nature of intelligence/cognitive abilities.” The percentage of respondents that studied group differences in ability and related topics was 55% for “group differences”; 33% for “consequences for economics, society, and culture”; 26% for “bias in ability tests”; 25% for “cross-cultural comparisons”; and 25% for “FLynn effect.” Respondents published an average of 47 papers (median = 20 papers), and reported high *h*-indexes (mean Scopus *h* = 17, median *h* = 11; mean Harzing *h* = 22, median *h* = 17). The published papers had passed peer review, a requirement for inclusion in Scopus *h*-index counts. The counts substantiate the high quantity and quality of research published by respondents.

One limitation of the study can be seen in the small sample and low response rates. The sample consisted of 71 respondents, which is small compared to Snyderman and Rothman's sample of 661 respondents (of 1020 invitations). In addition, self-selection of experts could have biased the results.

We attempted to increase response rates by using an Internet survey, emailing invitations (and reminders), and announcing the survey at intelligence conferences. Despite these measures, response rates were still low. The low response rates may be attributed to the length of the survey (which took about 40–90 min to complete), self-censorship, or fear of addressing a controversial subject (despite assurances of anonymity). The low response rates may also reflect a paucity of experts on intelligence and international differences in cognitive ability. There may be 20–50 scientists who study international differences in intelligence. Based on this estimate, the number of respondents (71 people) may exceed the number of scientists who study the topic! Because the aim of the survey was to obtain expert opinions on the research questions, our view is that participation of people with only tangential knowledge of the subject matter could distort answers more than low response rates attributable to self-selection by experts.

One researcher suggested in an email that only politically biased researchers would respond to the questionnaire, especially given its length. In contrast to the speculation, three researchers sometimes labeled as “conservative” in Internet articles and Wikipedia refused to participate due to lack of expertise, survey length, or disapproval of opinion surveys as a way of finding truth. Based on several comments of researchers with diverse political backgrounds we have no hint of biased participation or refusal of participation.

### Procedure

The Internet based survey was created with the free software LimeSurvey. Invitees received an invitation email with instructions on how to obtain a participation code, which could not be linked to the respondents' identities. Participants were told, “All questionnaires will be anonymously treated. No individual results will be published or given to other persons or the media. Results will be presented in an anonymous manner.” Only people who received a participation code could participate. After 2 weeks, invitees who had not responded automatically received a reminder email.

### Statistical analysis

Experts could assign a percentage to all 15 factors (causes) for each question. Logically, the percentages across all factors should sum to 100%. However, experts did not always rate all factors, and their ratings did not always sum to 100%. To address variation in percentages across participants (and questions), we analyzed only responses from experts who rated at least 5 of 15 factors or whose ratings summed to at least 75%. The sum of the ratings of 5 factors usually explained more than the half of the international differences. In addition, we replaced missing ratings with 0%, and standardized the sum of the ratings (within each question) to 100%. Replacing missing values with a score of 0% was based on the assumption that experts who did not rate a factor judged that factor as having no influence on ability differences.

The adjustments corrected for biases across raters and questions. For example, the average sum of items (i.e., factors) for the first global question (i.e., causes of cross-national differences) was 440%. With the corrections, the average sum of items for the first global question (and all other questions) was 100%. Without the corrections, the average sum of items for each question would deviate from 100%, and would prevent comparisons across questions.

## Results

### Causes of general cross-national differences

Seventy-one experts rated possible causes of cross-national differences in cognitive ability based on psychometric IQs and student assessment studies (e.g., PISA, PIRLS, TIMSS). Genes were rated as the most important cause (17%), followed by educational quality (11.44%), health (10.88%), and educational quantity (10.20%) (Table 1). The sum of both education factors yielded the highest rating (21.64%). Of all factors, genes had by far the largest standard deviation (*SD* = 23.85; all other factors, *SD* < 10), indicating disagreement about the importance of genetic influences. Only 5 of 71 experts (7%) who responded to the genetic item thought that genes had no influence. If non-responses to the genetic item are converted to 0% (4 additional experts), 13% of experts doubted any genetic influence. The frequency of zero-percentage-ratings was larger for genes than for culture or education (about 1%), but experts who believed that genes had no influence were a minority: Around 90% of experts believed that genes had at least some influence on cross-national differences in cognitive ability.

**Table 1:** **Means for each question and scale**.

	**Culture**	**Genes**	**Education quantity**	**Education quality**	**Wealth**	**Health**	**Geography**	**Current climate**	**Politics**	**Modernization**	**Sampling error**	**Test knowledge**	**Discrimination**	**Test bias**	**Migration**
Cross-national	7	**17**	10	**11**	9	**11**	3	2	5	7	4	6	2	3	3
Finland	10	**15**	**13**	**19**	9	10	2	2	6	6	2	3	1	1	3
East Asia	14	**20**	**17**	**15**	5	5	2	1	5	5	2	5	1	1	2
Sub-Sah. Africa	8	**19**	**12**	**12**	9	**12**	3	3	7	6	2	4	1	2	1
South Europe	**12**	**17**	11	**15**	8	6	3	3	9	6	2	4	1	1	3
Arab-Muslim	**15**	**17**	13	**14**	5	7	2	2	8	7	2	4	1	1	1
Latin America	11	**16**	**13**	**15**	8	8	2	2	7	6	2	4	1	1	1
Israel	**14**	**20**	9	**11**	6	7	3	2	8	4	2	3	1	1	8
Jews in West	**16**	**28**	11	**13**	8	6	1	1	3	4	3	3	2	1	2
Roma	**15**	**21**	14	**15**	7	7	1	1	3	6	1	3	2	1	2
Muslim immig.	**17**	**23**	12	**13**	6	5	1	1	4	5	2	3	2	3	2
Single average	**14**	**20**	**14**	**15**	7	7	2	2	6	5	2	3	1	1	2

*Scale between 0 (no influence) and 100 (explains all). Values are rounded to the nearest integer (better for reading and comparison) and may not sum to 100% (per row, due to rounding error). Respondents per row, beginning with “Cross-national” and ending with “Muslim immigration,” are as follows: N = 71, 62, 64, 60, 51, 52, 48, 46, 55, 49, and 46. The three most important factors per row are printed in bold. The bottom row (“Single average”) is the average rating across all single countries, regions, and groups (excluding cross-national general ratings in the first row)*.

Items with lower percentages (< 10%) included wealth, culture, and modernization (7–9%). Methodological bias factors (sampling error, test knowledge, test bias) were rated as less important (3–6%, together 11.78%).

### Causes for the ability levels of single countries and groups

The *Finnish miracle* refers to the top student test results in Finland (Simola, 2005). The top results in Finland have been attributed to progressive educational methods. However, traditional factors and declines in them (e.g., discipline and teacher authority) have been associated with recent drops in test results (Sahlgren, 2015). In the current study, experts attributed the top results in Finland to high educational quality (19.11%), followed by genetic-evolutionary factors (14.89%) and educational quantity (12.71%). The two educational factors combined totaled 31.87%. Educational quality was rated higher in Finland than in any other country.

*East Asian countries* attain high student test results in nearly all student assessment and psychometric IQ studies. Early test results from East Asia were attributed to better and more efficient instruction (Stigler et al., 1999), culture (Confucian achievement orientation; Helmke and Hesse, 2002), and evolutionary-genetic factors (Lynn, 1990; Kanazawa, 2004; Rushton, 2004). In the current study, experts attributed the high results in East Asia to genes (19.89%), education (quantity: 16.87%, quality: 14.72%), and culture (14.49%). Compared to other countries and regions, East Asia had the strongest ratings for educational quantity and test knowledge.

*sub-Saharan Africa* has the lowest student test results (in participating countries), a finding confirmed by psychometric IQs and Piagetian tasks (Lemos, 1974; Hallpike, 1978; Rindermann, 2013; Rindermann et al., 2014b). Similar to discussions of low intelligence in the US, the findings for sub-Saharan Africans are highly contentious, especially when factors other than the environment are examined (e.g., Segerstråle, 2000; Nyborg, 2003). Factors that have been implicated in the low results include health, wealth, evolution, political problems (corruption affecting educational means), modernization, and education (e.g., Lynn, 1990; Glewwe and Kremer, 2006). Measurement issues were also mentioned (e.g., Wober, 1969; Wicherts et al., 2010). In the current study, experts attributed the low results in sub-Saharan Africa to genetic-evolutionary factors (18.58%), followed by educational quality (12.27%), health (11.73%), and educational quantity (11.60%). The two educational factors together had the strongest rating (23.87%), and health had a high rating compared to other regions and countries (12%). While genes were rated as the most important single factor, there was considerable diversity of opinion: 10 of 60 experts gave genes a rating of zero (17%), and the standard deviation in ratings for genes was the highest of all factors (*SD* = 24.88; all other factors: *SD* < 10). Similar to other countries and regions, sub-Saharan Africa had low ratings for discrimination and methodological problems (sampling error, test bias, test knowledge).

*Southern Europe* attains low student test results compared to Central, West, and Northern Europe, and to North America and Australia/New Zealand^4^. The low results have been implicated in recent economic events (e.g., European debt crisis; Vanhanen, 2013). In the current study, experts attributed the low results in Southern Europe to genes (16.78%), educational quality (15.07%), and culture (12.28%). Compared to other countries and regions, Southern Europe had the highest rating for political factors (9.38%).

*Arab-Muslim countries* have low student test results and psychometric IQs, and also low levels of educational and scientific achievements, compared to other regions and countries (e.g., Arab Human Development Report; UNDP (United Nations Development Programme) Arab Fund for Economic Social Development, 2003). Even Arab students in engineering (which draws high ability students) achieved only average test results in a recent study (Rindermann et al., 2014a). In the current study, experts attributed the low results in Arab-Muslim countries to genetic-evolutionary factors (17.09%), followed by culture (14.70%), educational quality (14.41%), and educational quantity (13.00%). Compared to other regions, low modernization received the highest rating for Arab-Muslim countries (6.58%), a finding consistent with the Arab Human Development Report (UNDP (United Nations Development Programme) Arab Fund for Economic Social Development, 2003).

*Latin America* lags behind other regions in student assessment results, a finding that has been attributed to culture (e.g., Harrison, 2006). In the current study, experts attributed the low results in Latin America to genetic (16.42%) and educational factors (quality: 15.44%, quantity: 13.46%). The two educational factors combined had the strongest rating (28.90%). Culture received a moderate rating (11.34%), a finding similar to other regions (Table 1).

*Israel* is a puzzling case due to its relatively low test results compared to Jews in the West. The question was: “Results in Israel are lower than results in North-Western-Middle Europe. Why?” Lynn (2011) attributed the low results in Israel to diverse ethnic groups (e.g., Ashkenazim, Sephardim, Mizrahim, Ethiopian Jews, Palestinian Arabs), some of which lower the overall ability level. In the current study, experts attributed the Israeli results to the usual factors (genes: 20.49%; educational quality: 10.69%; educational quantity: 8.94%), with genes being rated slightly more important than both educational factors combined (19.63%). Compared to other countries and regions, education had the lowest rating, whereas immigration had the highest rating (8.20%).

*Jews in the West* show high student test results and also exceptional intellectual, scientific, and cultural achievements (e.g., Haag, 1969; Lipset and Raab, 1995). In the current study, experts attributed the high test results of Jews to genes (28.36%), which were rated more strongly than the two educational factors combined (quantity: 10.79%, quality: 13.38%; combined: 24.17%). Genetic factors had the highest rating compared to all other groups and countries. However, as in the cross-country analyses, there was disagreement about the importance of genes, with variability in ratings being higher for genes than for other factors (*SD* = 33.10; all other factors: *SD* < 16).

*Roma* (Romani people, gypsies with different subgroups) are the lowest scoring long-term natives in Europe (e.g., Cvorovic, 2014). The low test results have been attributed to low levels of education, health, wealth, and modernization, as well as discrimination and genetic factors (Rushton et al., 2007; Cvorovic, 2014). The expert survey revealed no special pattern of results. Experts attributed the low results of the Roma primarily to genetic factors (21.35%), followed by culture (15.16%) and education (quality: 14.73%, quantity: 14.15%). The two educational factors combined were seen as most influential (28.88%).

*Immigrants from the Middle East* (Arabian and Muslim countries) generally show low student test results and psychometric IQs, which are a third to one standard deviation lower than other groups (e.g., te Nijenhuis and Flier, 2001; Dronkers et al., 2012). The low test results of Middle Eastern immigrants have been attributed to culture, education, language, and discrimination (Dronkers et al., 2012). In the current study, experts attributed the low test results primarily to genetic factors (23.01%) and culture (17.26%). Discrimination was rated as relatively unimportant for Middle Eastern immigrants (2.27%) and for other groups and countries (1.25%). The two educational factors combined were rated as most important (quality: 13.30%, quantity: 12.14%, combined: 25.44%).

### Causes for the ability levels of single countries and groups—average ratings

The strongest rated factor across all countries, regions, and groups was genes-evolution (19.72%), followed by educational quality (14.69%), culture (13.71%), and educational quantity (13.60%; Table 1, last row). These four factors were followed by health (7.32%), wealth (7.27%), politics (5.56%), and modernization (4.90%). Other factors received weak ratings (e.g., geography, current climate, migration, discrimination), including methodological factors (e.g., sampling error, test knowledge, test bias). Nature and nurture compared, genes were rated as the most important factor, but the two educational factors combined (quality and quantity), and all environmental factors together (e.g., education, culture, wealth, health), were rated more strongly than genes.

Cultural factors were rated as more important for single countries than for general cross-country comparisons (13.71 vs. 7.35%). In addition, compared to cross-country comparisons, single countries showed higher ratings for genes (19.72 vs. 16.99%), educational quantity (13.60 vs. 10.20%), and educational quality (14.69 vs. 11.44%). In contrast, single countries showed lower ratings for wealth, health, modernization, and test bias (total of sampling error, test knowledge, and test bias: 6.26% single-country vs. 11.78% cross-country), suggesting that general cross-country comparisons reflect global impressions that may not hold for single countries.

### Comments

There were few entries in the comment sections. Two experts noted that the results for Israel depended on a mix of ethnicities and climate, without detailed explanation. Other experts noted that strong discrimination may be a cause of Jewish high intelligence, and that the causes of international ability differences are interconnected. Another expert stressed that Finns have the “fastest simple visual reaction times of any contemporary Western population,” and referred to a publication.

## Discussion

Experts on cognitive ability rated possible causes of international differences in student assessment and psychometric IQ test results. Ratings were obtained for cross-national differences and single countries using a percentage scale ranging from 0 to 100% (higher percentages = more important). The expert survey revealed important results: Methodological factors (sampling error, test bias, test knowledge) were weakly rated (cross-national: 11.78%; single countries' average: 6.26%), as was discrimination (cross-national: 2.10%, single countries: 1.23%). In contrast, educational factors (quality and quantity) and genes were strongly rated. The low ratings for methodological factors suggest that international assessments were perceived to be valid indicators of cognitive ability and cross-country patterns.

Experts rated the two *educational factors* together (quantity and quality) as the most important cause of international differences in cognitive ability (cross-national: 21.64%, single countries' average: 28.29%). Weaker ratings were given for environmental factors such as health (cross-national: 10.88%, single countries: 7.32%), wealth (cross-national: 8.96%, single countries: 7.28%), modernization (cross-national: 7.19%, single countries: 4.91%), and politics (cross-national: 4.77%, single countries: 5.56%). The sum of all these *environmental factors* explained more than half of the international ability differences (cross-national: 53.44%; single- countries' average: 53.36%).

The relative importance of environmental factors does not mean that genetic factors were seen as irrelevant. Based on expert opinions, the *genetic-evolutionary factor* was the single most important cause of international differences in cognitive ability (cross-national: 16.99%, single-country: 19.72%): Experts attributed about one-sixth to one-fifth of international ability differences to genes. While the rated impact of genes was remarkable, it was still well below the rated impact of environmental factors (around 50%). In addition, disagreement among experts (based on *SD*s in ratings) was much higher for genes than for environmental factors. Other factors such as geographic and climate differences were rated as negligible influences on international ability differences. Immigration was rated as important only for Israel.

While both camps, the nature as well as the nurture camp, are supported by the results of the expert survey, the scientifically more interesting result is the support for genetic explanations. Because environmental theories are rarely questioned in research, their corroboration by an expert survey is not astonishing. More important is the support for the frequently hotly disputed genetic explanations. Assuming that the survey is representative of expert opinions, genetic factors should receive more attention in future research and public debates. To fairly consider different hypotheses, future research should incorporate procedures (e.g., rules for methods of argumentation) that reduce zeitgeist or political pressures that may bias responses on controversial issues (e.g., Segerstråle, 2000; Jussim et al., 2015).

This finally leads to some limitations of the presented study: The main limitation can be seen in the low response rate (20%). Although low response rates can produce unreliable results, low response rates are less problematic in the current study, which was designed to solicit *informed* opinions about ability differences. Because experts who responded to the survey had conducted research in relevant areas (e.g., cognitive ability, group differences, test bias, cross-cultural comparisons, FLynn effects), they were in a good position to provide informed opinions of ability differences. Thus, although response rates were low, the responses were likely to reflect the opinions of people who had expertise on the topic being evaluated.

A related limitation is self-selection. Only a small number of experts responded to the invitation, and the responses of these experts may be unrepresentative and biased. Such problems are common to surveys, but might be less relevant in the current study, which was designed to attract experts in a narrow domain. (There are perhaps no more than 50 experts in the world who specialize in international ability differences.) The experts who completed the survey reported that they had expertise in intelligence and group differences. This suggests that the experts had self-selected into the survey based on relevant expertise, which should increase the validity of opinions. Based on our experience, we suggest that future surveys more carefully preselect who is invited to participate. In the current study, our aim was to recruit a broad sample of experts, including experts who might be reluctant to respond for various reasons, but not to exclude anybody who perceives him- or herself as an expert. In retrospect, our recruitment procedure was probably too broad, yielding many invitations but few responses, which lowered response rates.

A third limitation concerned the measurement of factors that cause international ability differences. Different factors were measured with different numbers of items. Education was measured with two items, environmental factors with 11 items, and genetics with one item. To better estimate the importance of nature and nurture, a single binary question could be added to future surveys (e.g., “Which is more influential, genetic or environmental factors?”). In addition, the survey did not assess indirect influences. Additionally, genetic effects can be included in the rated environmental aspects health, wealth, institutional features and migration or in non-rated aspects of personality (Dawkins, 2008/1982; Spolaore and Wacziarg, 2013; Woodley et al., 2014). Finally, culture can influence genetics via consanguineous marriages (e.g., Tadmouri et al., 2009).

Further research is needed to identify the facets of the factors that contributed to expert opinions. The facets may include different aspects of educational quality (e.g., student discipline or class size) or different types of genes (e.g., generalist genes or genes for specific abilities).

It should be noted that an expert survey is both an *opinion instrument* and a possible *indicator of truth*. As an opinion instrument, the survey would be biased if the selection of experts were biased. In the current study, experts were selected if they had published on intelligence or attended an intelligence conference, increasing the likelihood that the experts had an informed opinion. The other aspect of an expert survey—that it is an indicator of truth—cannot be answered by this study. A single empirical study can contradict expert opinion, and the results of the current survey must be validated in future empirical research.

Finally, the expert survey did not address *the interdependence of factors*. Factors related to cognitive ability may influence each other in complex ways. For example, culture may influence education, genes, health, and cognitive ability, which in turn may influence wealth and economic development (e.g., Rindermann et al., 2013, 2015). These interdependencies could be examined using path models and longitudinal datasets. Although expert surveys cannot replace empirical research, the current survey provides a snapshot of expert opinions, gives a balanced appraisal of current trends, and highlights targets for future studies (e.g., educational quality and genetic factors).

## Author contributions

All three authors developed the study concept. DB programmed the web-based questionnaire and carried out the survey. DB and HR performed the data analysis. DB drafted an initial version of the manuscript. HR and TC provided revisions. All authors approved the final version of the manuscript for submission.

### Conflict of interest statement

The authors declare that the research was conducted in the absence of any commercial or financial relationships that could be construed as a potential conflict of interest.

## Footnotes

^1^PISA (Programme for International Student Assessment, internationally, since 2000), PIRLS (Progress in International Reading Literacy Study, since 2001), and TIMSS (Trends in International Mathematics and Science Study, since 1995).

^2^The conversion transforms the SAS-scale (*M* = 500, *SD* = 100) in developed countries to an IQ scale (*M* = 100, *SD* = 15). We assumed an increase of 35 SAS points, or 3 IQ points per year of schooling (Winship and Korenman, 1997; Rindermann, 2011).

^3^The comparability of student assessments and intelligence tests was analyzed in Rindermann and Baumeister (2015) and Rindermann (2007). On the comparability of student assessment and intelligence test scales see Rindermann and Baumeister (2015) and Rindermann (2007).

^4^However, the picture is not uniform. The northern regions of Italy and Spain have results similar to those of other Western countries (OECD, 2007; Lynn, 2010, 2012).
