# Supplementary material for: Survey of Expert Opinion on Intelligence: Causes of International Differences in Cognitive Ability Tests
Source: Front Psychol. 2016 Mar 23;7:399. doi: 10.3389/fpsyg.2016.00399 (PMC4804158; doi:10.3389/fpsyg.2016.00399)
Supplement: Supplementary file 1 [file DataSheet1.docx]

# Appendix

The questions on international differences and their causes using the original question numbering.

40a On causes of cross-national differences, what do you think are the reasons for large differences in cognitive ability and intelligence studies (including PISA, TIMSS etc.)?

40b One of the countries with the highest scores on international assessments (especially PISA) is Finland. Why do you think this is the case?

40c The region with the highest scores on international assessments is East-Asia (Japan, Korea, the Chinas). Why do you think this is the case?

40d The region with the lowest scores on international assessments is sub-Saharan Africa. Why do you think this is the case?

40e Results in Southern Europe are lower than results in North-Western-Middle Europe. Why?

40f Results in the Arabian-Muslim world are lower than results in North-Western-Middle Europe. Why?

40g Results in South America are lower than results in North-America. Why?

40h Results in Israel are lower than results in North-Western-Middle Europe. Why?

40i Results of Jews in the Western world are at the top. Why?

40j Results of Roma (gypsies) in Europe are comparatively low. Why?

40k Results of immigrants from the Middle East (Arabian and Muslim countries) in the Western world are not at the top. Why?

**For every single question the instruction was:**

“To address this question, please rate the factors below in importance (it is difficult to clearly distinguish them because they are somewhat connected, so please give only a rough estimate).”

**To be rated casual factors were (in this order):**

Culture (religion, tradition, etc.); Genes (evolution); Education (quantity); Education (quality); Wealth; Health; Geography; Current climate; Politics; Modernization; Sampling error etc.; Test knowledge; Discrimination (-/+); Test bias (-/+); Migration (-/+)

**The rating scale was:**

01-05%; 06-10%; 11-15%; 16-20%; 21-25%; 26-30%; 31-35%; 36-40%; 41-45%; 46-50%; 51-55%; 56-60%; 61-65%; 66-70%; 71-75%; 76-80%; 81-85%; 86-90%; 91-95%; 96-100%; No answer.
